# Analytical methods in strigolactone research

**DOI:** 10.1186/s13007-020-00616-2

**Published:** 2020-05-29

**Authors:** Rostislav Halouzka, Sanja Ćavar Zeljković, Bořivoj Klejdus, Petr Tarkowski

**Affiliations:** 1 Centre of Region Haná for Biotechnological and Agricultural Research, Department of Phytochemistry, Faculty of Science, Palacky University, Šlechtitelů 27, 78371 Olomouc, Czechia; 2 Centre of Region Haná for Biotechnological and Agricultural Research, Department of Genetic Resources for Vegetables, Medicinal and Special Plants, Crop Research Institute, Šlechtitelů 29, 78371 Olomouc, Czechia; 3 Central European Institute of Technology, Mendel University in Brno, Zemědělská 1, 613 00 Brno, Czechia; 4 Department of Chemistry and Biochemistry, Mendel University in Brno, Zemědělská 1, 613 00 Brno, Czechia

**Keywords:** Strigolactones, Isolation, Determination, GC–MS, LC–MS/MS, HR-DART-MS, DESI-MS

## Abstract

Strigolactones (SLs) are important plant hormones that are produced via the carotenoid biosynthetic pathway and occur at extremely low concentrations in various plant species. They regulate root development, play important roles in symbioses between higher plants and mycorrhizal fungi, and stimulate germination of plant–parasitic *Orobanche* and *Striga* species. Chemical analysis is central to research on the biochemistry of SLs and their roles in developmental biology and plant physiology. Here we summarize key issues relating to the identification and quantification of SLs isolated from plant tissues and exudates. The advantages and drawbacks of different protocols used for strigolactone analysis are discussed, and guidelines for selecting a procedure that will minimize losses during isolation and purification prior to final analysis are proposed. Hyphenated techniques suitable for SL analysis such as GC–MS and LC–MS/MS are also discussed, and newer ambient techniques such as HR-DART-MS and DESI-MS are highlighted as tools with considerable potential in SL research. A key advantage of these methods is that they require only simply sample preparation.

## Background

Strigolactones (SLs) are a poorly characterized group of plant hormones [1], although they have been known for over 60 years because of their interactions with parasitic weeds such as *Orobanche*, *Striga* and *Phelipanche*. They occur in diverse plant species, ranging from mosses to higher plants such as *Pinus* sp. and *Eucalyptus* sp. [2–5]. The first SLs to be described were strigol and its acetate, both of which were isolated from cotton and named after the plant genus *Striga*, which is parasitic on cotton [2, 6].

As plant hormones, SLs regulate developmental processes including the induction of secondary growth, acceleration of leaf senescence, stimulation of internode growth, and root elongation. They also inhibit axillary bud outgrowth and the formation of adventitious and lateral roots [7]. Additionally, SLs serve as signaling molecules with important roles in the induction of hyphal branching in arbuscular mycorrhiza (AM) and stimulating seed germination in parasitic plants [1, 8]. Recent findings indicate variation in the biological activity of SLs. For instance, orobanchol is highly active towards AM fungi but is a weaker stimulator of parasitic seed germination in *Striga hermonthica* than its biosynthetic precursor *ent*-2′-*epi*-5-deoxystrigol [9].

**Fig. 1:**
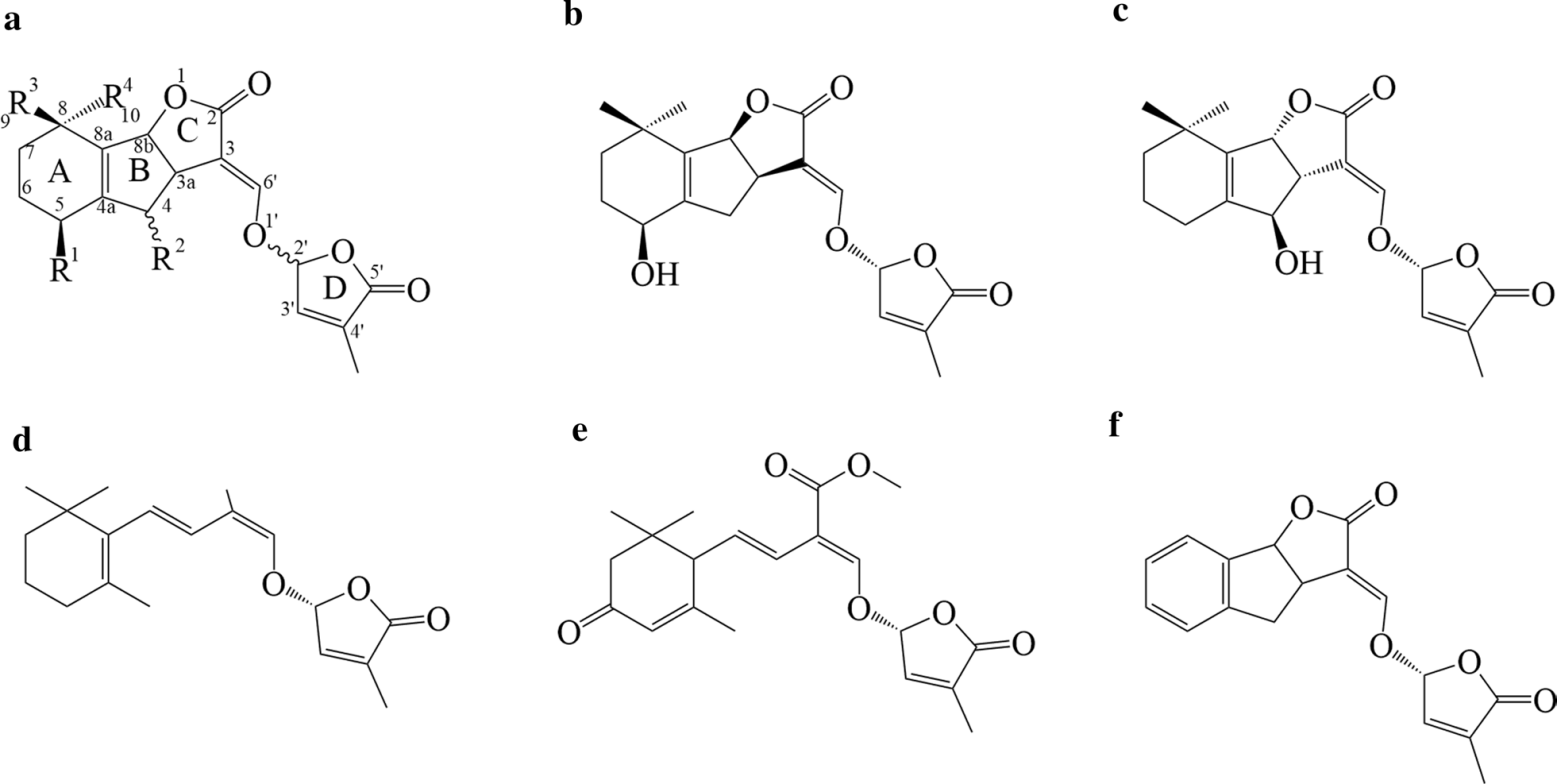
Chemical structures of selected strigolactones. **a** General structure; **b** (+)-Strigol; **c** (+)-Orobanchol; **d** Carlactone; **e** Heliolactone; **f** Synthetic SL analogue (GR24)

SL biosynthesis occurs primarily in the roots [3], from where SLs are either secreted into the rhizosphere or transported to the shoots [10–12]. They are synthesized via the carotenoid biosynthetic pathway [1]. In structural terms, SLs consist of a tricyclic lactone (ABC moiety) and a methylated butenolide (ring D) coupled via an enol ether linkage. The enol ether is unstable and easily cleaved or hydrolysed even under mild conditions [13]. Ring D is a characteristic feature of all naturally occurring SLs (Fig. 1), [14]. Minor modifications of the ABC moiety such as methylation, acetylation, or hydroxylation influence the compound’s biological activity. Enzymatic hydroxylation can occur on the AB rings; hydroxylation at the C-4, C-5, and C-9 positions results in the formation of orobanchol, strigol, and sorgomol, respectively. Conversely, no SLs hydroxylated on the C or D rings have yet been reported [9, 15]; such structures would be quite unstable. There are also non-canonical SLs such as heliolactone (Fig. 1e) from sunflowers [16] and lotuslactone from *Lotus japonicus* [17]. These compounds lack the A-, B-, or C- ring but retain the enol-ether-D ring moiety, which is essential for the biological activities of SLs.

Chemical analysis of small molecules, including plant hormones, is central to studies on plant development and host-microbe interactions. In particular, information on hormone levels is essential for researchers working on any kind of hormone-regulated process. The analysis of phytohormones such as strigolactones is very challenging because the endogenous concentration of the target molecules in plant tissues are often very low (on the order of pg/g fresh weight), so the presence of much more abundant compounds can cause significant interference. These problems can be particularly severe if the interfering compounds have similar structures and/or physicochemical properties to the target analytes. Other factors that complicate hormonal analysis include the high complexity of plant matrices and the limited chemical, light, and thermal stability of many target compounds [18–20]. Proper purification is necessary to obtain reliable data, and simplification of purification protocols often intensifies matrix effects, making final analysis very difficult or even impracticable [21, 22]. The most popular technique for final analysis of phytohormones over the last decade has been liquid chromatography-tandem mass spectrometry, which combines a high-resolution chromatographic system with sensitive and acceptably selective mass spectrometric detection [20].

This review summarizes the advantages and disadvantages of isolating SLs from root tissue and exudates, presents noteworthy findings related to their qualitative and quantitative chemical analysis, and offers some future perspectives on research in this area.

## Isolation and purification

Sample preparation is an essential step in any chemical analysis of plant metabolites, especially phytohormones. SLs occur mainly in root exudates and root tissues [11, 23] but the SL profiles of root exudates and roots differ, probably because of the different biological roles of individual compounds. Their isolation from root exudates is limited by the presence of inorganic salts in the medium. Extracts containing such salts are incompatible with the chromatographic methods used for final analyses due to their harmful effects on the detectors. In addition, extraction requires the processing of relatively large volumes of media (typically litres). Therefore, the capacity of the solvent and/or sorbent used must be carefully considered when analyzing root exudates.

Conversely, isolating and purifying SLs from root tissues requires pre-concentration of the target compounds, sample desalting, and also removal of interfering substances. In this case, the main unwanted contaminants are organic compounds with physicochemical properties similar to the target analytes.

SLs are produced in very low quantities, on the order of 15–30 pg/plant/day [18, 24, 25]. It is therefore essential to minimize losses during isolation and purification in order to maximize recovery of the target compounds over the sample preparation process. Isolating SLs from plants is very laborious, and it is generally impossible to guarantee that the quantities obtained will be sufficient to determine the structure of previously uncharacterized SLs [14, 18]. The first SL isolation procedures required over 25,000 plants, but the use of modern chromatographic and spectrometric methods has significantly reduced the number of plants needed [26, 27]. Over the last two decades, the quantity of plant material required for SL profiling has fallen from tens of kilograms (e.g. Yokota et al. [27]) to grams or even milligrams (e.g. Charnikova et al. [28] or Rial et al. [29]), mainly because of advances in instrumentation, but also because of more efficient sample isolation and purification procedures.

The most common procedures for SL isolation from root exudates are based on adsorption on charcoal [30–32]. Many rather similar protocols for isolating and purifying SLs from root tissue have been presented [11, 33, 34]; that developed by Yoneyama et al. [34] is generally accepted as the standard protocol, but was recently modified by Boutet-Mercey et al. [35]. They ground the plant material before extraction, while Yoneyama et al. [34] extracted intact plant tissue. In addition, Boutet-Mercey et al. [35] purified the extract via SPE. More details and the differences between these two approaches are described in the following section.

## Isolation of SLs from root exudates

Plant exudates are complex mixtures of bioactive phytochemicals containing both low- and high-molecular weight compounds that are important for plant adaptation and defence [36, 37]. Exudates are generally secreted by plant root hairs, calli, and suspension cells [36]. The collection of exuded bioactive phytochemicals is a non-destructive process that can be repeated several times to obtain higher quantities of the desired molecules [38, 39]. Plant seedlings for SL production are usually grown in hydroponic culture systems. In these systems, seeds are sterilized and germinated, then once the seedlings are 2 days old, they are transferred into a strainer with a sheet of gauze linked to a slightly larger container containing various volumes of tap water or nutrient solution [40, 41]. Tap water can typically be used as the medium for the first 3–5 days to acclimatize the plants, after which a growth medium containing nutrients is applied. The medium can be used throughout the cultivation process, and because SL production and exudation are sensitive to nutrient availability [40–43], the cultivation timeframe can be tuned. Three different types of cultivation media are widely used today: most European groups use Hoagland medium, but Japanese researchers prefer Tadano and Tanaka medium or a modified Long Ashton nutrient solution [28, 44, 45]. These cultivation media contain various inorganic salts, which may bind to target compounds. For instance, higher concentrations of phosphate (≥ 5 mM) in the cultivation media negatively affect both the production and stability of SLs in root exudates. Rial et al. [29] analyzed both exudates and root tissues from tomato plants grown under –P and +P conditions, and observed significantly lower SL concentrations in samples grown under +P conditions. We found that phosphate and some other nucleophiles promoted the degradation of the SL synthetic analog, GR24 [13]. Phosphate is a good example of an inorganic ion influencing both SL biosynthesis in plants and their stability in aqueous solutions.

There are two common approaches for collection/extraction of SLs from root exudates: (A) combined collection with charcoal followed by SPE (solid phase extraction) and (B) direct LLE (liquid/liquid extraction) of target compounds from the media [23, 30, 40, 43] (Table 1). Combined collection yields relatively low recoveries—typically, > 20% (Halouzka, unpublished results)—and the selectivity of adsorption on charcoal is limited by the large quantities of hydroponic solution that must be passed through the sorbent. Adsorbed root exudates are eluted with acetone, evaporated to dryness, re-dissolved in water, and then extracted with ethyl acetate (EtOAc) via LLE [30]. EtOAc is the preferred extraction solvent for SLs due to its moderate polarity and low toxicity. However, the use of freshly distilled solvent is strongly recommended because residual acetic acid degradates SLs. The EtOAc extracts are then washed with 0.2 M K_2_HPO_4_ (pH 8.3) to obtain a neutral fraction. Finally, the extract is dried over anhydrous MgSO_4_ or NaSO_4_ and concentrated *in vacuo* [40, 43].

**Table 1:** Overview of analytical methods for strigolactones

Analytical technique	Column	Mobile phase composition	Analysis time	Matrix	Extraction solvent	Purification	Strigolactone	References
LC-ESI-MS/MS	ODS (C18), Mightysil RP-18 (2 × 250 mm, 5 µm)	A—H_2_O B—MeOH	34–60 min	Tissue	EtOAc	LLE	5-Deoxystrigol 7-Oxoorobanchyl acetate 7α-Hydroxyorobanchyl acetate Didehydro-orobanchol Fabacyl acetate Methyl zealactonoate Orobanchol Orobanchyl acetate Solanacol Sorgolactone Sorgomol Strigol Strigyl acetate	[5, 15, 18, 30, 32, 40, 41, 43–45, 48, 49]
				Exudates	–	Adsorption on charcoal and elution with acetone; LLE with EtOAc		
	L-Column2 ODS (2.1 × 50 mm, 2.0 μm)	A—H_2_O B—MeOH	22 min	Exudates	–	Adsorption on charcoal and elution with acetone; LLE with EtOAc	5-Deoxystrigol Orobanchol Orobanchyl acetate	[25]
	COSMOSIL 2.5C18-MS-II (100 × 2.0 mm, 2.5 µm)	A—H_2_O B—MeOH	20 min	Exudates	EtOAc	CC (silica)	4-Deoxyorobanchol 5-Deoxystrigol Carlactone Carlactonoic acid Heliolactone	[16, 52]
	ACE Excel 1.7 C18 (100 mm × 2.1 mm, 1.7 μm)	A—0.1% FA/H_2_O B—0.1% FA/ACN	10.5 min	Exudates	–	Adsorption on C18 and elution with acetone	5-Deoxystrigol 7-Oxoorobanchyl acetate Fabacyl acetate Orobanchol Orobanchyl acetate Solanacol Strigol	[29]
				Tissue	EtOAC			
	ACQUITY BEH C18 (100 × 2.1 mm, 1.7 mm)	A—0.1% FA/H_2_O B—ACN	12 min	Exudates	–	Adsorption on C18 and elution with acetone	Zealactones 1-5	[28]
		A—H_2_O B—ACN	19 min	Tissue	EtOAc	CC (silica)	Fabacyl acetate Orobanchol Orobanchyl acetate	[35]
		A—H_2_O B—MeOH	9 min	Exudates	–	Adsorption on C18 and elution with acetone	5-Deoxystrigol 7-Oxoorobanchyl acetate Fabacyl acetate Orobanchol Orobanchyl acetate Solanacol Strigol	[33]
	ACCLAIM 120C18 (2.1 mm × 250 mm, 5 μm)	A—0.1% FA/H_2_O B—0.1% FA/ACN	40 min	Exudates	EtOAc	C18 SPE	5-Deoxystrigol Fabacyl acetate Orobanchyl acetate Orobanchol Solanacol	[23]
				Tissue	EtOAc	CC (silica)		
	Kinetex C18 (2.1 × 150 mm, 2.6 μm)	A—0.1% AcA/H_2_O B—0.1% AcA/ACN	23 min	Tissue	EtOAc	CC (silica)	5-Deoxystrigol	[34]
GC-MS	DB-5 (4 m × 0.25 mm)	He	27 min	Exudates	–	Adsorption on XAD-4 and elution with EtOAc; CC on Sephadex LH-20	Methyl zealactonoate Orobanchol	[27, 47, 48]
				Tissue	EtOAc			

*AcA* acetic acid, *FA* formic acid, *LLE* liquid–liquid extraction, *CC* column chromatography, *SPE* solid phase extraction

**Fig. 2:**
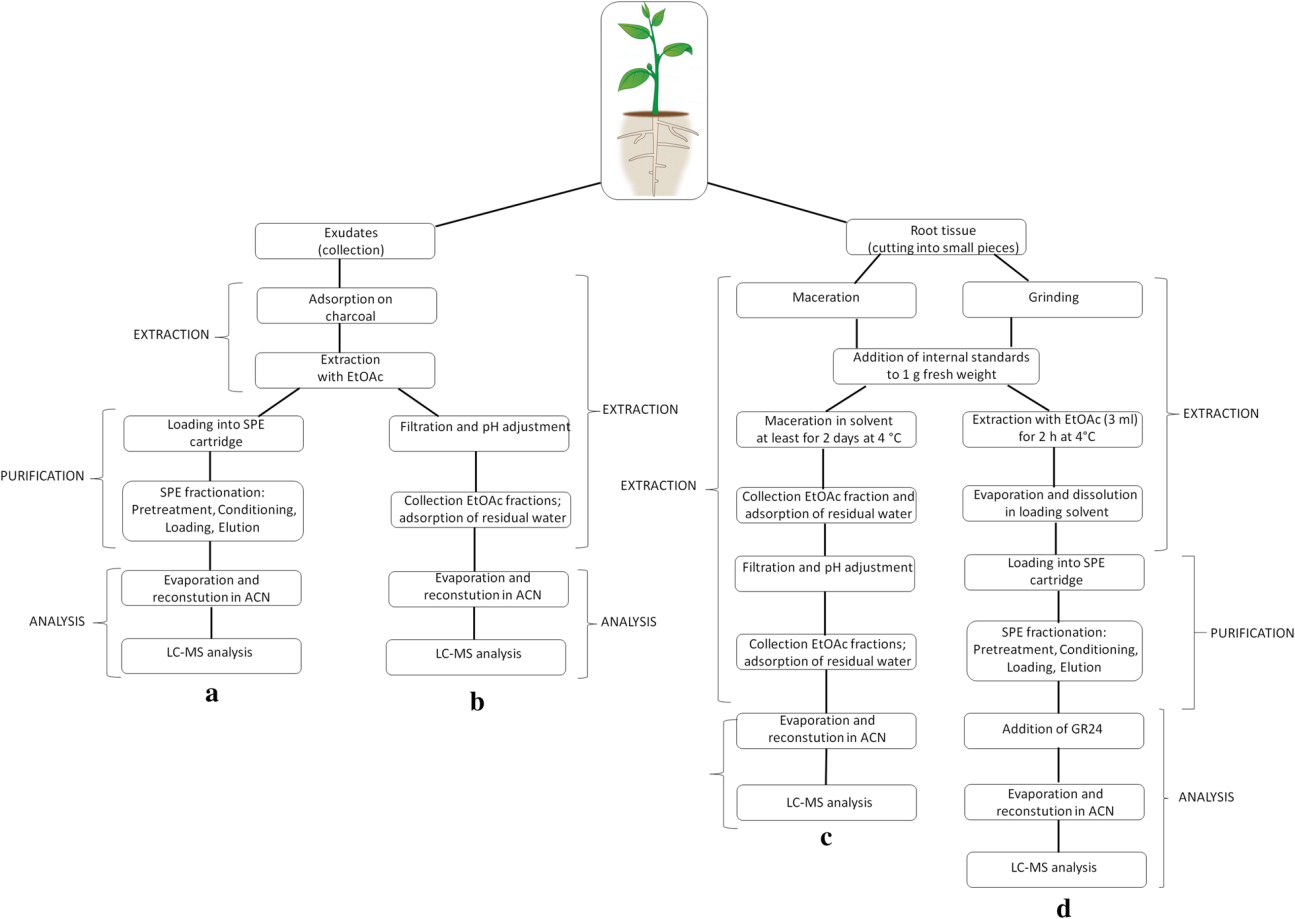
A common scheme for isolating and purifying SLs from plant exudates and root tissue. (**a** [31]; **b** [40]; **c** [34]; **d** [35])

The LLE approach involves directly extracting SLs from the cultivation media without prior adsorption on a sorbent. The collected root exudates are extracted repeatedly with an equal volume of EtOAc [43], then the extracts are combined and neutralized (Fig. 2B). Collection and extraction are repeatable processes that can be performed over several days [31]. Most SLs characterized to date were isolated from root exudates [18, 28, 30, 32, 33].

A third way of isolating SLs from exudates was presented by Rial et al. [29], who analyzed concentrated exudates without pretreatment after dissolution in MeOH. This resulted in successful quantification even though the nucleophilicity of MeOH makes it a suboptimal solvent for SLs: 5-deoxystrigol reportedly has a half-life of 1.5 days in 3% MeOH [8, 13].

## Isolation of SLs from root tissues

The transfer of target analytes from root tissues to organic solvents requires either maceration or extraction of the ground tissue. Maceration involves submerging the intact tissue in freshly distilled solvent for a few days, while extraction involves first homogenizing the plant tissue with a vibration mill or mortar and pestle. To avoid enzymatic or chemical degradation, the tissue must be cooled to 4 °C during extraction. The efficiency of maceration/extraction depends on the target molecule’s polarity and subcellular localisation. Plant hormones are usually associated with other compounds such as phenolics, lipids, and proteins. The extraction solvent must therefore minimize the extraction of interfering substances and not affect the stability or chemical properties of the target compounds [34, 46].

Yoneyama et al. [34] established a standard SL isolation protocol (Fig. 2c) that was successfully applied to sorghum root tissue. This protocol involves macerating small pieces of roots or shoots from 2 to 4 week-old plants in EtOAc. Maceration should not be performed for more than 3 days due to the water content of the plant tissue; water can easily decompose both SLs and EtOAc under slightly acidic conditions. Other measures to prevent potential analyte loss include pH neutralization, water removal, and omitting nucleophilic substances or solvents (MeOH). EtOAc and acetone are popular solvents for these processes. The final steps are similar to those for isolation from exudates, i.e. washing with K_2_HPO_4_, drying over anhydrous MgSO_4_ or Na_2_SO_4_, filtration, and evaporation under reduced pressure at temperatures below 35 °C. A very similar isolation protocol was described by López-Ráez et al. [33], who directly analysed EtOAc extracts of tomato tissues [29, 33].

An alternative approach was developed by Boutet-Mercey et al. [35], who combined the extraction of homogenized tissue with purification by SPE (Fig. 2d). Their protocol was tested and optimized for SLs isolated from garden peas (fabacyl acetate, orobanchyl acetate, and orobanchol). While other phytohormones are often purified by SPE using hydrophobic C18-type or polymer-type sorbents, silica is commonly used for SLs. This prevents the use of aqueous solvents, which has the additional benefit of improving SL stability and reducing evaporation times [20, 35]. Successful quantitative analysis of SLs from tissue samples requires both purification and fractionation. However, fractionation requires the handling of many fractions—more than ten in some cases. To generate these fractions, elution is performed stepwise while varying the composition of the eluent. The elution solvent is typically a mixture of EtOAc with heptane or hexane, in ratios ranging from 100:0 to 0:100 [31]. The optimized SPE protocol of Boutet-Mercey et al. [35] generates only four fractions (2 washing and 2 elutions). These authors also reported significant matrix effects for fractions containing higher amounts of EtOAc. A drawback of the SPE procedure is that it increases the total time required for analysis to over 24 h [29], which is problematic because the instability of SLs means that time is an important factor in their analysis. It is therefore necessary to strike a balance between extraction efficiency and stability. Based on experience and published results, we make the following recommendations for the use of endogenous SL and synthetic analogues such as GR24 in plant treatments [13, 31–33]:

- Stock solutions should be prepared using dry inert solvents such as DMF, acetone or acetonitrile, with a maximum storage temperature of − 20 °C;
- Aqueous solutions containing SLs must be used within 24 h;
- Methanol is not a suitable solvent for experiments with SLs;
- EtOAc is a good extraction solvent but must be redistilled before extraction; and
- Sample preparation (including SPE purification) should be fast and produce an appropriate number of fractions.

## Identification and quantification

### GC–MS

Gas chromatography (GC) is a very important method in phytohormone analysis, although it is often replaced by liquid chromatography because it requires the derivatization of non-volatile analytes. Nevertheless, Yokota et al. [27] showed that GC is a viable tool for studying SLs by using GC coupled with a mass spectrometer (MS) operating in electron ionization mode (EI) to identify strigol and orobanchol in red clover. They used a non-polar capillary column packed with 5% (phenyl)methylpolysiloxane, which was also used by Erickson et al. [47], and Xie et al. [48] (Table 1).

SLs are non-volatile and thermolabile compounds. Their volatility and stability in the injection port of a GC system (150–250 °C) can be improved by derivatization with tetramethylsilane (TMSi), which causes cleavage of the D-ring.

**Fig. 3:**
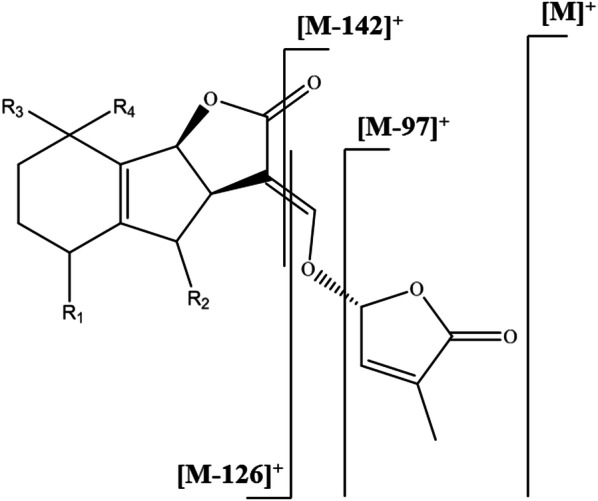
EI-MS fragmentation pattern for canonical SLs

Electron ionization in GC–MS analyzers is always performed at 70 eV, which is clearly too high for SLs, causing their molecular peaks to have very low intensities (approximately 4% of relative abundance). Based on the data summarized by Ćavar et al. [46], we propose that SLs exhibit the fragmentation pattern shown in Fig. 3 when ionized at 70 eV in a GC-EI-MS analyzer. The base peak for all SLs analyzed by GC-EI-MS is *m/z* 97, which corresponds to the cleaved hydroxymethyl butenolide, i.e. C_5_H_5_O_2_^+^. The peaks corresponding to the ABC-moiety and its fragments formed by cleavage of the enol ether [M-126]^+^ and further cleavage of the oxo-group from the lactone [M-142]^+^ have quite low intensities—at most 15% of relative abundance.

Since the introduction of HPLC coupled with triple quadrupole (QqQ) mass detectors for identification of SLs by Sato et al. [18], GC has largely served as a supporting method for confirming the spectral characteristics of SLs [47, 49].

### LC–MS

The most popular separation method for SLs is high-performance liquid chromatography (HPLC). Separation of SLs is usually performed on a reversed-phase stationary phase with C18 bonded silica columns. The most common mobile phases are mixtures of neutral or acidified water with methanol or acetonitrile (Table 1). In the early days of SL research, HPLC systems equipped with spectrophotometric detectors (UV or DAD) were used. The usefulness of such systems is limited by their sensitivity and the difficulty of interpreting the UV profiles of SLs—for example, strigol and orobanchol have the same chromophore [27, 50]. This problem is illustrated by the example of Siame et al. [50], who used UV detection and mistakenly reported that strigol occurred in maize (*Zea mays*). Two decades later, Jamil et al. [51] disproved this finding and showed that neither strigol nor any other canonical SLs occur in maize. Instead, several non-canonical SLs were detected in maize root exudates [28, 48, 51].

Traditional HPLC is being replaced by UHPLC (ultra-high performance liquid chromatography), which offers shorter analysis times and greater separation efficiencies. Routine run-times in UHPLC are typically less than 20 min (Table 1). Quantitation of SLs by UHPLC–MS/MS (tandem mass spectrometry) is typically performed using the multiple reaction monitoring (MRM) mode [3, 18, 52]. The value of this selective detection mode is demonstrated in a recently published paper by Rial et al. [29], who could not completely separate the isomeric compounds strigol and orobanchol by reversed-phase chromatography but were able to selectively distinguish them by monitoring the relevant MRM transitions.

It should be noted that most SLs are analyzed in positive ion mode but carlactonic acid is analyzed in negative ion mode (Table 2), [52]. Additionally, the choice of MRM transitions depends strongly on mobile phase composition and pH. Acidic conditions suppress formation of sodium or potassium adducts in favor of protonated quasi-molecular ions ([M + H]^+^). However, most authors focus on transitions of sodium adduct ions [M + Na]^+^, with the most abundant fragment corresponding to neutral loss of [M + Na − 97]^+^ [15, 18, 30, 33–35, 44, 49, 53]. This transition is monitored because all known SLs have identical D-ring moieties [3]. Table 2 provides an overview of canonical and non-canonical SLs with defined MRM transitions observed in LC–MS/MS experiments.

**Table 2:** Overview of *m/z* transitions for known canonical and non-canonical SLs

Strigolactone	[M + H]^+^	MRM1	MRM2	References	[M + Na]^+^	MRM1	MRM2	References
Orobanchol	347	347 > 205	347 > 97	[11]	369	369 > 272		[82]
Orobanchyl acetate	389	389 > 347	389 > 233	[11]	411	411 > 254	411 > 239	[49]
4-deoxyorobanchol	331	331 > 216		[52]				
7-oxoorobanchol					383	383 > 286		[44]
7-oxoorobanchyl acetate					425	425 > 268		[44]
7*α*-hydroxyorobanchol					385	385 > 288		[44]
7*α*-hydroxyorobanchyl acetate					427	427 > 270		[44]
7*β*-hydroxyorobanchol					385	385 > 288		[44]
7*β*-hydroxyorobanchyl acetate					427	427 > 270		[44]
Solanacol	343	343 > 97	343 > 183	[87]	365	365 > 268		[33]
Solanacyl acetate					407	407 > 250		[25]
Strigol					369	369 > 272		[18]
Strigyl acetate					411	411 > 254		[83]
Strigone					367	367 > 270		[31]
5-deoxystrigol	331	331 > 216	331 > 97	[88]	353	353 > 256		[84]
Sorgolactone	317	317 > 97		[23]	339	339 > 242		[83]
Sorgomol					369	369 > 272		[85]
Fabacyl acetate	405	405 > 231	405 > 97	[29]	427	427 > 219	427 > 242	[44]
Fabacol					385	385 > 288		[44]
Heliolactone	361	361 > 233	361 > 97	[16]				
Zealactone	377	377 > 345	377 > 97	[28]				
Avenaol	377	377 > 263	377 > 97	[89]				
Carlactone	303	303 > 97		[52]				
Calactonic acid*	331	331 > 113		[52]				
Methyl carlactonate	347	347 > 97		[86]				
Methoxy-5-deoxystrigol					383	383 > 286		[25]

*Carlactonic acid—precursor ion [M - H]^−^

## Future tools

### Flash chromatography

As demonstrated by the above discussion, there is still a need for a general and practical tool for SL isolation. Flash chromatography or extraction using monolithic sorbents could be attractive alternatives to SPE purification. Flash chromatography uses a hybrid medium with small silica gel particles (250–400 mesh size) that necessitate the application of positive pressure to force solvent through the column. Automated flash chromatography systems are multifunction devices that closely resemble HPLC systems: they have gradient pumps, sample injection ports, UV detectors, and inbuilt fraction collectors. They can be used to separate target analytes on scales ranging from a few mg to kilograms, and are much cheaper than preparative HPLC systems [54]. An alternative option is to use monolithic sorbents, whose pore systems enable their use as general tools for isolating SLs and separating their stereoisomers. Some monolithic silica sorbents have additional useful features such as high linear velocities, which increase separation efficiency and thus facilitate the isolation of pure isomers [55].

### Nuclear magnetic resonance

Mass spectrometric techniques are currently indispensable tools for SL detection. A wide range of techniques suitable for diverse applications are available. In recent years, several research groups studying SLs have used a TOF (time of flight) mass analyser together with a tandem mass spectrometry (MS/MS) setup that enables fragmentation of separated compounds and analysis of the resulting ionized fragments [28, 48].

An overlooked technique is LC coupled with on-line NMR, which enables real-time detection and detailed characterization of the eluting compounds, including determination of factors such as their stereochemical properties [56]. Unfortunately, its sensitivity and selectivity are much lower than those of MS methods, so it is mainly used for nonselective analysis. The sensitivity of NMR can be improved by using dynamic molecular polarization or cryo- and microprobes [57, 58]. However, even these refinements are insufficient to match the sensitivity and selectivity of MS-based methods, which can detect target analytes in the pmol to fmol range [59, 60]. On the other hand, NMR offers very high reproducibility and requires minimal sample preparation [57]. It is therefore often used in metabolomic fingerprinting studies that focus on identifying and quantifying compounds associated with drug metabolism and food intake, and for NMR-based metabolomics in phytochemical studies [59–61].

### MS ambient techniques

**Fig. 4:**
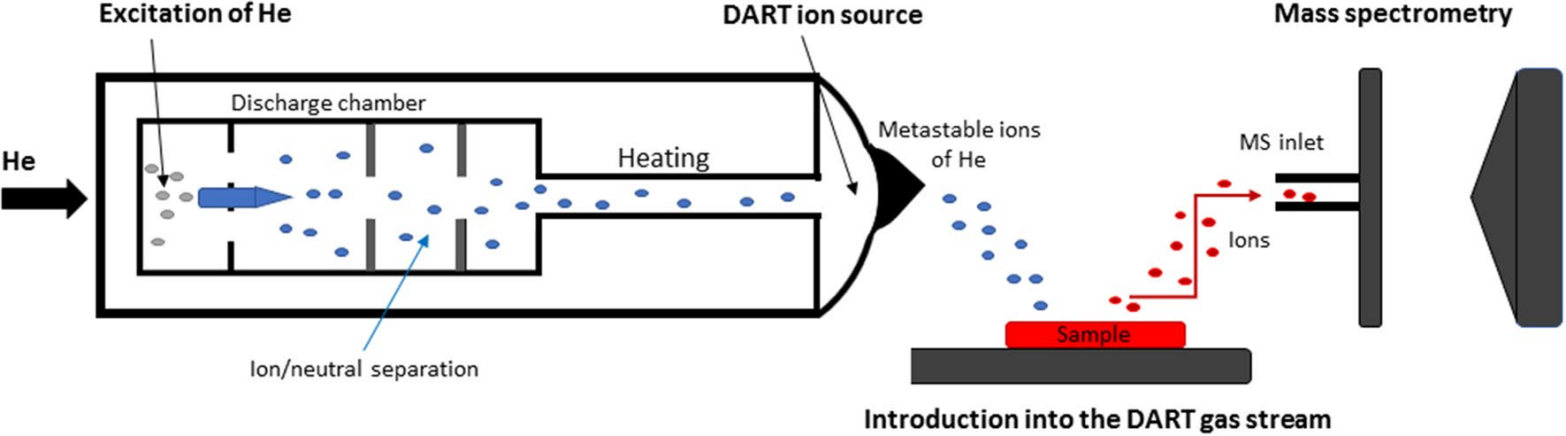
Operating principles of HR-DART-MS. Adopted from [80]

Other important modern methods are ambient techniques (AT) such as DART (direct analysis in real time) and DESI (desorption electrospray ionization) [62, 63], which can be used to determine the spatial distribution of target compounds in a sample. These relatively new mass spectrometric techniques use an ion source located outside the mass analyser and are suitable for a wide range of low molecular mass compounds. A huge advantage of DART is that it requires minimal or even no sample preparation; small tissue samples or crude extracts can be introduced directly into the ion source [64], (Fig. 4). However, the reproducibility of AT techniques can be limited by problems resulting from outer ionization. AT techniques could potentially serve as the basis of a general fast identification method suitable for detecting SLs in various plant tissues (stems, roots, and leaves). However, many issues remain to be addressed including problems with sample shrinkage (due to losses of water), which changes the nature of the tissue surface [65–68]. Also, each plant organ has a unique structure and thus requires separate process optimization. Coupling DART with HRMS (high resolution mass spectrometry) could make it possible to determine the mass (m/z) of any compound in a plant tissue sample with relatively high mass accuracy (below 1 ppm).

**Fig. 5:**
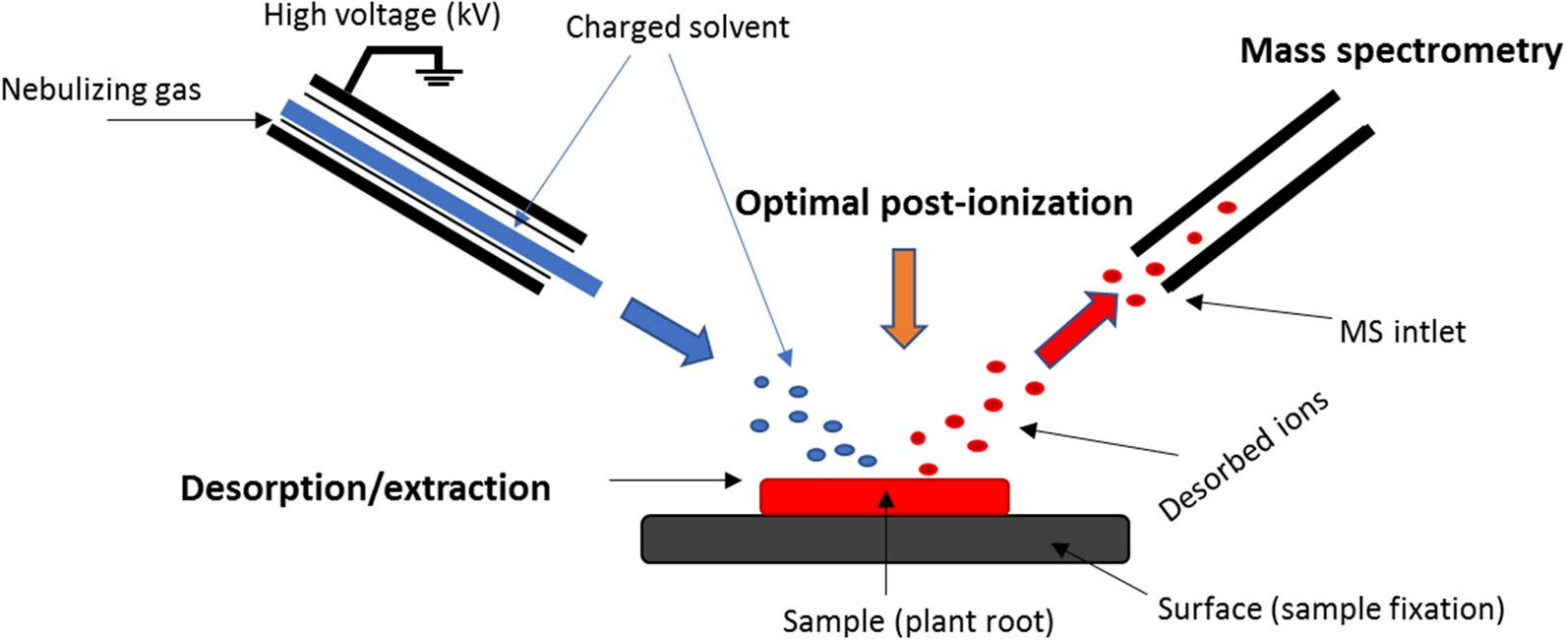
Operating principles of DESI-MS. Adopted from [81]

DESI is a mass spectrometry imaging (MSI) technique that provides information about the spatial distribution of target analytes [69] by combining electrospray ionization (ESI) and desorption ionization. It is particularly suitable for low molecular weight compounds such as SLs. The sample to be analysed is fixed or imprinted (Fig. 5) onto the solid surface of a plate, which can be a TLC plate or a plate made from porous Teflon, paper, glass, or plastic [69–71].

The key advantages of MSI (DESI) are that it requires minimal sample manipulation and no separation steps. However, it is very sensitive to matrix effects, which can significantly affect the observed spatial distribution of analytes. In particular, physical properties (roughness on the surface of the analysed sample) can profoundly affect the analysis and the reliability of results obtained using this method [72–74]. Rejšek et al. [74] recently showed that DESI-MS can also be applied to non-planar samples by using an ion source with a laser triangulation system. This upgraded DESI setup obviates the need for chemical pretreatment of samples or sample preparation by imprinting. Moreover, it preserves the native spatial distribution of SLs and other target compounds in the sample.

Another important MSI technique is laser ablation electrospray ionization (LAESI), which was introduced in 2007 [75]. This method also achieves high spatial resolutions (below 1 μm) and has been used to perform quantitative analyses of tissue samples from several plants. For example, it was used to study arginine levels in *A. cepa* bulbs [76]. However, like DESI, it is limited by being much less well-developed than MALDI (matrix-assisted laser desorption/ionization). While DESI and LAESI cannot yet match the resolution achieved with MALDI, they are potentially powerful tools for analysing plant metabolomes and could thus help answer a number of outstanding questions about chemical organization in plants [68, 77, 78]. Plant hormones occur in minute quantities, so that sensitive analytical tools are required for their analyses. The major limitation of MS ambient techniques is their low sensitivity, which could be improved by increasing either the ionization efficiency or the sensitivity of ion detection. To assess the potential of DART- and DESI-MS we are currently finalizing a method paper that discusses this issue.

## Conclusions

Chemical analysis is central to research on the biochemistry of SLs and their roles in plant development and physiology. Before performing any such analysis, it is essential to know at least the main chemical properties of the target analytes, such as their structures, stability under certain conditions, and chemical reactivity, as well as the levels at which they exist in the tissues of interest. This review summarized the different methods that have been used to isolate and purify SLs from root tissues and exudates, and to identify the SLs present in the resulting isolates. The advantages and drawbacks of each method have been highlighted, which should be valuable information for plant scientists seeking to study these phytohormones. Additionally, we have presented guidelines for protocol selection that should help minimize losses during isolation and purification prior to final analysis.

Qualitative and especially quantitative analyses of SLs are needed to clarify their roles in regulating plant development and their interactions with other phytohormones. Of the techniques available for this purpose, LC–MS/MS continues to be the most generally useful and widely used [79]. However, more recently developed highly sensitive analytical methods such as HR-DART-MS and HR-DESI-MS can provide information that cannot be obtained using LC-MS/MS, such as data on the spatial distribution of target analytes. As such, they could be valuable complementary tools for semi-quantitative analysis of SLs. Additionally, these methods require little or no sample preparation, enabling rapid analysis with minimal risk of analyte degradation.

## Data Availability

Not applicable
